# Targeting the PI3K/Akt signaling pathway in pancreatic β‐cells to enhance their survival and function: An emerging therapeutic strategy for type 1 diabetes

**DOI:** 10.1111/1753-0407.13252

**Published:** 2022-02-22

**Authors:** Inah Camaya, Sheila Donnelly, Bronwyn O'Brien

**Affiliations:** ^1^ School of Life Sciences, Faculty of Science The University of Technology Sydney Ultimo New South Wales Australia

**Keywords:** FhHDM‐1, PI3K/Akt, type 1 diabetes, β‐cell, 1型糖尿病, β‐细胞, PI3K/AKT, FhHDM‐1

## Abstract

Type 1 diabetes (T1D) is an autoimmune disease caused by the destruction of the insulin‐producing β‐cells within the pancreas. Islet transplantation represents one cure; however, during islet preparation and post transplantation significant amounts of β‐cell death occur. Therefore, prevention and cure of T1D is dependent upon the preservation of β‐cell function and the prevention of β‐cell death. Phosphoinositide 3‐kinase (PI3K)/Akt signaling represents a promising therapeutic target for T1D due to its pronounced effects on cellular survival, proliferation, and metabolism. A growing amount of evidence indicates that PI3K/Akt signaling is a critical determinant of β‐cell mass and function. Modulation of the PI3K/Akt pathway, directly (via the use of highly specific protein and peptide‐based biologics, excretory/secretory products of parasitic worms, and complex constituents of plant extracts) or indirectly (through microRNA interactions) can regulate the β‐cell processes to ultimately determine the fate of β‐cell mass. An important consideration is the identification of the specific PI3K/Akt pathway modulators that enhance β‐cell function and prevent β‐cell death without inducing excessive β‐cell proliferation, which may carry carcinogenic side effects. Among potential PI3K/Akt pathway agonists, we have identified a novel parasite‐derived protein, termed FhHDM‐1 (*Fasciola hepatica* helminth defense molecule 1), which efficiently stimulates the PI3K/Akt pathway in β‐cells to enhance function and prevent death without concomitantly inducing proliferation unlike several other identified stimulators of PI3K/Akt signaling . As such, FhHDM‐1 will inform the design of biologics aimed at targeting the PI3K/Akt pathway to prevent/ameliorate not only T1D but also T2D, which is now widely recognized as an inflammatory disease characterized by β‐cell dysfunction and death. This review will explore the modulation of the PI3K/Akt signaling pathway as a novel strategy to enhance β‐cell function and survival.

## Graphical abstract

**Highlights**

- Phosphoinositide 3‐kinase (PI3K)/Akt signaling is an emerging promising therapeutic target for diabetes.
- Modulation of the PI3K/Akt signaling pathway, via protein and peptide‐based biologics, parasite‐derived molecules, plant extracts, or through microRNA interactions, will regulate the processes that determine the function of β‐cells and the fate of β‐cell mass.
- Therapeutic approaches targeting PI3K/Akt signaling will have broad‐reaching applications for the prevention of type 1 diabetes, the preservation of islets prior and post transplantation, and the treatment of type 2 diabetes

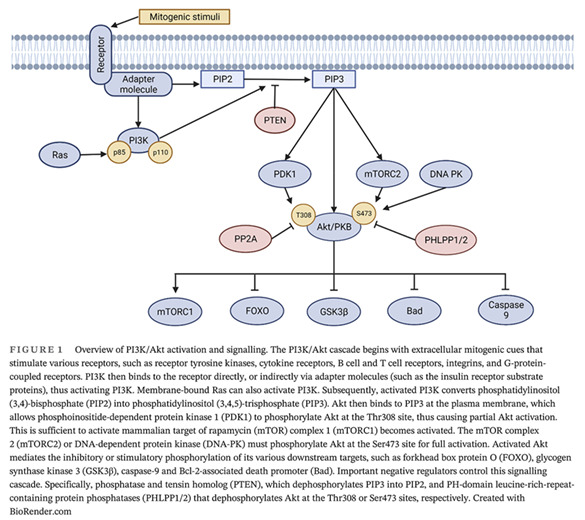

## Abstract (zh)

1型糖尿病(T1D)是一种自身免疫性疾病，由胰腺内分泌胰岛素的β细胞破坏引起。胰岛移植是一种治疗方法，然而，在胰岛准备和移植后期间，大量的β细胞会发生死亡。因此，T1D的防治有赖于β细胞功能的保护和β细胞死亡的预防。磷脂酰肌醇3‐激酶(PI3K)/Akt信号通路对细胞存活、增殖和代谢有显著影响，是治疗T1D的有效靶点。越来越多的证据表明，PI3K/AKT信号是决定β细胞质量和功能的关键因素。通过直接(使用高度特异的蛋白质或基于多肽的生物制剂、寄生虫的产物和植物提取物的复杂成分)或间接(通过microRNA相互作用)调节PI3K/Akt通路，从而调节β‐细胞过程，可最终决定β‐细胞团的结局。一个重要的考虑是确定特定的PI3K/AKT途径调节剂，这些调节剂可以增强β细胞的功能，防止β细胞死亡，而不会诱导β细胞过度增殖，这可能会带来致癌副作用。在潜在的PI3K/Akt途径激动剂中，我们已经发现了一种新的寄生虫衍生蛋白，称为FhHDM‐1(肝片吸虫蠕虫防御分子1)，能有效刺激β‐细胞的PI3K/Akt途径，增强功能并防止死亡，而不像其他几个已发现的PI3K/Akt信号刺激因子那样同时诱导增殖。因此，FhHDM‐1将为针对PI3K/Akt途径的生物制剂设计提供信息，以预防/改善T1D和T2D，T2D现在被广泛认为是一种以β细胞功能障碍和死亡为特征的炎症性疾病。本文将探讨PI3K/Akt信号通路的调控作为提高β细胞功能和存活的新策略

## 1 INTRODUCTION

Type 1 diabetes (T1D) is an autoimmune disease caused by the destruction of the insulin‐producing β‐cells within the pancreatic islets of Langerhans.^1^ Multiple immune cell subsets contribute to the elimination of β‐cells. Neutrophils, macrophages, and T helper 1 (TH1) and TH17 CD4^+^ T cells produce pro‐inflammatory cytokines and other cytotoxic mediators, which can directly destroy β‐cells and/or prime autoreactive CD8^+^ cytotoxic T cells. The initiation and amplification of immune responses against β‐cell autoantigens occurs during an extensive, yet silent, preclinical period, which precedes the onset of clinical symptoms (notably hyperglycemia) by months to years. Initiation of the destructive autoimmune processes likely begins early in life, concurrent with waves of β‐cell neogenesis (formation of β‐cells from non‐β‐cell precursors), proliferation, and apoptosis, which occur during a physiological phase of neonatal pancreatic remodeling. Perturbations in the balance between these proliferative and apoptotic processes within β‐cells can have pathogenic consequences. Specifically, increased rates of β‐cell apoptosis (perhaps coupled with impaired clearance of dying cells by macrophages) can lead to the generation of autoantigens,^2^, ^3^ which activate autoreactive pro‐inflammatory TH1 and TH17 cells along with cytotoxic T cells. Following the destruction of 80% to 90% of the β‐cell population, the patient becomes irreversibly hyperglycemic and must rely on exogenous insulin to survive. However, insulin injections cannot mimic the minute‐to‐minute glucose responsiveness of β‐cells. Therefore, the patient is subject to episodes of hypoglycemia and hyperglycemia, which increase morbidity and mortality.^1^ Preservation of the survival and metabolic activities of β‐cells is required to prevent and cure T1D.

## 2 TARGETING β‐CELLS TO TREAT T1D

The progressive loss and dysfunction of pancreatic β‐cells is the key pathogenic process of T1D. Testament to this, for several months up to a year after diagnosis, over half of T1D patients experience a “honeymoon period” in which the remaining β‐cell mass produces sufficient insulin to maintain normoglycemia. However, this residual β‐cell population is ultimately destroyed by autoreactive immune cells and their mediators. Similarly, after islet transplantation, recurrent autoimmunity eliminates the allograft in the absence of chronic immunosuppression, which carries multiple adverse side effects, including β‐cell death. Additionally, significant amounts of β‐cell destruction occur during the preparation of islets for transplantation. This demands the availability of two to three cadaver pancreata to revert a single patient to normoglycemia, and reversion is often transient due to subsequent β‐cell destruction. Therefore, prevention and cure of T1D is dependent upon the preservation of β‐cell function and the prevention of β‐cell death.

Despite the knowledge that β‐cell death underpins T1D, none of the currently used antidiabetic agents directly target the maintenance of endogenous β‐cell mass. This is because the therapeutic focus has been on blocking the pro‐inflammatory autoimmune responses to halt the β‐cell destructive autoimmune sequelae in T1D patients and those predisposed to T1D. These strategies rely upon modulation of immune cell populations and do not directly impact β‐cells to maintain function and/or prevent apoptosis. Testament to these shortcomings, diabetes reversal is not achieved, protection of residual β‐cell mass is short‐term, global immune suppression is induced, and multiple adverse side effects are experienced.^4^, ^5^, ^6^ With an understanding that it is the dysregulation in the rates of β‐cell proliferation vs apoptosis early in life that serve as pivotal initiators of autoimmunity and that T1D development proceeds as autoreactive immune cells perpetuate the destructive responses,^7^, ^8^, ^9^ regulation of these processes in β‐cells could effectively maintain sufficient β‐cell mass to halt T1D development. Given the limitations of current T1D therapies, it is crucial to investigate more direct strategies to preserve β‐cell mass and/or promote β‐cell function.

One pathway that is emerging as a central process to the development, and therefore prevention, of T1D is the phosphoinositide 3‐kinase (PI3K)/Akt signaling pathway. PI3K/Akt signaling has long been recognized as a major regulator of important cellular processes, such as survival, proliferation, lipid metabolism, protein synthesis, glucose homeostasis, and apoptosis. It is now apparent that this holds true for the target cells destroyed in T1D, the pancreatic β‐cells. Indeed, a growing body of evidence indicates that PI3K/Akt signaling is a critical regulator of β‐cell processes that determine the fate of β‐cell mass, including proliferation, survival, metabolism, and apoptosis. This review will explore the modulation of the PI3K/Akt signaling pathway as a novel strategy to preserve β‐cell function and avoid β‐cell death to prevent/ameliorate T1D.

## 3 The PI3K/AKT signaling pathway

PI3K belongs to a family of kinases that catalyze the phosphorylation of inositol lipids. There are three PI3K classes (denoted I, II, and III), which are differentiated by structure and substrate specificity. Class I is further subdivided into class IA PI3K and class IB PI3K. The former are dimers of a p110 catalytic subunit (isoforms: p110α, p110β, and p110δ) and a p85 regulatory subunit (isoforms: p85α, p85β, p55α, p55γ, and p50α), while the latter are dimers of a p100γ catalytic subunit with a p101 or p87 regulatory subunit.^10^, ^11^ In the context of β‐cell survival and function in T1D, class I PI3K are the most relevant and will herein be referred to as “PI3K.”

**FIGURE 1:**
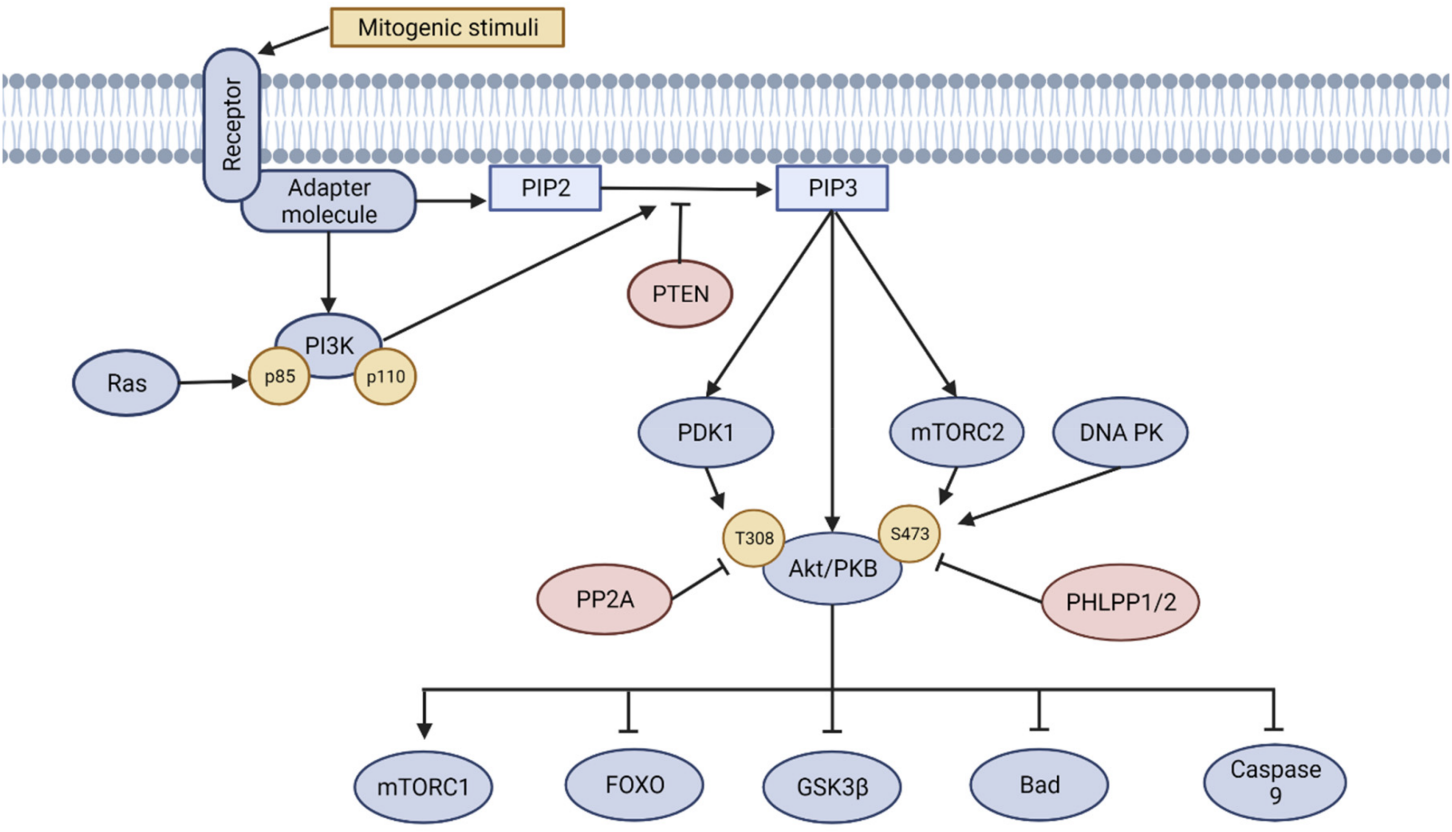
Overview of PI3K/Akt activation and signalling. The PI3K/Akt cascade begins with extracellular mitogenic cues that stimulate various receptors, such as receptor tyrosine kinases, cytokine receptors, B cell and T cell receptors, integrins, and G‐protein‐coupled receptors. PI3K then binds to the receptor directly, or indirectly via adapter molecules (such as the insulin receptor substrate proteins), thus activating PI3K. Membrane‐bound Ras can also activate PI3K. Subsequently, activated PI3K converts phosphatidylinositol (3,4)‐bisphosphate (PIP2) into phosphatidylinositol (3,4,5)‐trisphosphate (PIP3). Akt then binds to PIP3 at the plasma membrane, which allows phosphoinositide‐dependent protein kinase 1 (PDK1) to phosphorylate Akt at the Thr308 site, thus causing partial Akt activation. This is sufficient to activate mammalian target of rapamycin (mTOR) complex 1 (mTORC1) becomes activated. The mTOR complex 2 (mTORC2) or DNA‐dependent protein kinase (DNA‐PK) must phosphorylate Akt at the Ser473 site for full activation. Activated Akt mediates the inhibitory or stimulatory phosphorylation of its various downstream targets, such as forkhead box protein O (FOXO), glycogen synthase kinase 3 (GSK3β), caspase‐9 and Bcl‐2‐associated death promoter (Bad). Important negative regulators control this signalling cascade. Specifically, phosphatase and tensin homolog (PTEN), which dephosphorylates PIP3 into PIP2, and PH‐domain leucine‐rich‐repeat‐containing protein phosphatases (PHLPP1/2) that dephosphorylates Akt at the Thr308 or Ser473 sites, respectively. Created with BioRender.com

Generally, PI3K is activated by the binding of extracellular growth ligands to their cognate receptors, including receptor tyrosine kinases (RTK), cytokine receptors, B cell and T cell receptors, integrins, and G‐protein‐coupled receptors. After ligand/receptor interaction, PI3K binds to the receptor, either directly (via its regulatory subunit) or indirectly (through adapter molecules, such as the insulin receptor substrate [IRS] proteins), thereby causing PI3K activation (Figure 1). Additionally, direct or indirect activation of PI3K can be mediated by the catalytic subunit (p110) in conjunction with small GTPases, notably Ras and Ras‐related protein Rab‐5A.^12^, ^13^

Once activated through its catalytic subunit, PI3K converts phosphatidylinositol (3,4)‐bisphosphate (PIP2) into phosphatidylinositol (3,4,5)‐trisphosphate (PIP3) to which Akt (also known as protein kinase B [PKB]) then binds. This allows phosphoinositide‐dependent protein kinase 1 (PDK1) to phosphorylate Akt at its Thr308 site. This event causes partial activation of Akt. Subsequently, the mammalian target of rapamycin complex 1 (mTORC1) becomes activated and interacts with multiple downstream substrates. For full activation, Akt must be phosphorylated at the Ser473 site, which is catalyzed by mTOR complex 2 (mTORC2) or DNA‐dependent protein kinase (DNA‐PK). Once fully activated, Akt can induce further substrate‐specific phosphorylation events in both the cytoplasm and nucleus.^11^, ^14^

The precise function of Akt is determined by the specific context of its phosphorylation, including factors such as isoforms and different phosphorylation sites. There are three conserved isoforms of Akt: Akt1/PKBα, Akt2/PKBβ, and Akt3/PKBγ. Akt1 and Akt2 are expressed ubiquitously, while Akt3 is highly expressed in nervous tissue. Evidence from mouse models suggests that these isoforms are associated with distinct functions in various cells.^11^, ^15^ Furthermore, Akt activation is modulated by phosphorylation at different sites, aside from the major sites Thr308 and Ser473. Indeed, isoforms Akt1, Akt2, and Akt3 have 31, 22, and 18 known potential phosphorylation sites, respectively.^16^ It is the large number of potential phosphorylation sites in Akt, and its ability to catalyze the phosphorylation of multiple substrates, that facilitates the involvement of fully activated Akt in multiple and varied cellular events.

Activated Akt can have an inhibitory or stimulatory effect, depending on the target substrate and the consequent phosphorylation events, thereby resulting in the modulation of several cellular processes, such as glucose metabolism, apoptosis, proliferation, gene transcription, and migration.^11^, ^14^ For instance, Akt exerts an inhibitory effect on forkhead box protein O (FOXO) to prevent its pro‐apoptotic and catabolic activities.^11^, ^17^ Akt also represses glycogen synthase kinase 3 (GSK3β), leading to inhibition of GSK3β‐mediated apoptosis and glycogen synthase.^18^ Akt can also directly inhibit the activity of the pro‐apoptotic proteins caspase‐9 and Bcl‐2‐associated death promoter (Bad), thereby promoting cell survival. Collectively, activated Akt modulates the functions of its substrates to promote cell proliferation, survival, and/or metabolism while simultaneously inhibiting apoptosis.^11^, ^19^

This pro‐survival pathway is tightly controlled by key negative regulators, such as the phosphatase and tensin homolog (PTEN), which antagonizes Akt signaling by dephosphorylating the products of PI3K activity (ie, reverting PIP3 to PIP2). Protein phosphatase 2 (PP2A) and PH‐domain leucine‐rich‐repeat‐containing protein phosphatases (PHLPP1/2) also suppress Akt activity by dephosphorylating Akt at the Thr308 or Ser473 sites, respectively.^11^, ^14^ The tight regulation of PI3K activities enables the optimal balance between pro‐survival/proliferative/metabolic and apoptotic events to be achieved.

## 4 TRANSGENIC MOUSE MODELS PROVIDE EVIDENCE THAT PI3K/AKT SIGNALING MODULATES β‐CELL MASS AND FUNCTION

Experiments using transgenic murine models, in which specific components of the PI3K/Akt pathway have been overexpressed in β‐cells, have provided direct evidence that PI3K/Akt signaling is a critical determinant of β‐cell mass and function. Overexpression of the constitutively active form of Akt1 (CA‐Akt) in β‐cells in vivo induced a significant increase in β‐cell size and total islet mass, with resultant improved glucose tolerance, which protected mice against streptozotocin (STZ)‐induced diabetes (multiple low doses of 40 mg/kg body weight for five consecutive days, which causes autoimmune diabetes).^20^ Given that the rate of β‐cell proliferation remained unchanged, it was concluded that disease protection was associated with an Akt‐mediated preservation of β‐cell mass and increased metabolic activity, specifically the anabolic processes that drive cell growth. Similar maintenance of islet mass and glucose tolerance were observed in an equivalent CA‐Akt mouse model, which also exhibited resistance to β‐cell death ordinarily rapidly induced by higher doses of STZ.^21^ Interestingly, in contrast to the original CA‐Akt model, protection against diabetes was attributed to enhanced β‐cell proliferation (and putatively β‐cell neogenesis) resulting in increased islet mass, which counteracted the rates of β‐cell destruction.^21^ Thus, modulation of the PI3K/Akt signaling, via increased expression levels of CA‐Akt, positively regulated β‐cell mass and function to prevent diabetes. The enhancement of β‐cell proliferation, while effective in the short term to negate β‐cell destruction, must be monitored to ensure that pancreatic carcinogenesis does not ensue.

Likewise, expression of constitutively active epidermal growth factor receptor (CA‐EGFR; a RTK that preferentially stimulates PI3K/Akt signaling), at a time coincident with neonatal pancreatic remodeling, increased rates of β‐cell proliferation in mice. This positive effect on β‐cell growth was not observed in adult mice, suggesting that the modulation of β‐cell mass dynamics, and prevention of T1D development, is most effectively achieved early in life.^22^ This is an interesting observation, given that the physiological wave of neonatal islet remodeling is juxtapositioned with the initiation of autoimmunity in susceptible animal models, such as nonobese diabetic (NOD) mice, with concomitant increased rates of β‐cell apoptosis and trafficking/infiltration of autoreactive immune cell populations to the islets (insulitis).^2^, ^3^, ^7^ Nevertheless, in adult mice, CA‐EGFR expression improved glucose tolerance and significantly inhibited β‐cell apoptosis following either a single high dose (200 mg/kg body weight) or multiple low doses (50 mg/kg body weight daily for five consecutive days) of STZ, thereby conferring partial protection against diabetes development. Further, islets isolated from diabetes‐resistant (CA‐EGFR overexpression) animals were resistant to the cytotoxic effects of the pro‐inflammatory cytokines (IL‐1β, tumor necrosis factor [TNF], and IFNγ) that are primarily responsible for β‐cell destruction in T1D. The protective mechanism was likely attributable to PI3K/Akt‐mediated inhibition of the pro‐apoptotic protein Bcl‐2‐interacting mediator of cell death (BIM), which facilitates cytokine‐induced β‐cell apoptosis during T1D development.^22^

Indirect modulation of the PI3K/Akt pathway has also been achieved by expression of the caspase‐3‐generated RasGAP N‐terminal fragment (fragment N), a molecule that exerts an anti‐apoptotic effect via activation of Ras‐PI3K‐Akt signaling in various cell types,^23^ including insulin‐secreting cells. This is supported by in vivo studies utilizing a transgenic NOD mouse model in which fragment N was expressed specifically within the β‐cells. These mice exhibited slower progression to hyperglycemia and overt diabetes as compared to non‐transgenic NOD controls. Further in situ studies of islets isolated from these transgenic animals at 16 weeks of age (when significant rates of β‐cell apoptosis would normally be occurring) showed that fragment N expression was associated with reduced numbers of apoptotic β‐cells as compared to controls. This anti‐apoptotic effect was attributable to the activity of Ras, which in turn activated PI3K/Akt signaling .^24^

Consistent with these observations, deletion of the key negative regulator of the PI3K/Akt pathway PTEN in β‐cells has also been shown to provide protection against STZ‐induced diabetes.^25^ In vitro studies using the murine β‐cell line β‐TC‐6 showed that silencing of PTEN conferred resistance to cytokine‐induced apoptosis and partially reversed Akt inhibition.^26^ Collectively, these observations provide compelling evidence that activation of the PI3K pathway in β‐cells has the potential to positively modulate β‐cell function to prevent diabetes development.

## 5 MODULATORS OF PI3K/AKT Signaling Are Potential Therapeutics

### 5.1 Proteins and peptide‐based biologics

The use of protein or peptide‐based agents is now being extensively studied due to their ability to stimulate β‐cell proliferation/function and/or inhibit β‐cell apoptosis, specifically through modulation of PI3K/Akt signaling (Table 1). Treatment of NOD‐derived NIT‐1 β‐cells with erythropoietin (EPO) suppressed pro‐inflammatory cytokine‐induced apoptosis, and improved insulin secretion, in a PI3K/Akt‐dependent manner.^27^ Similarly, the protein Wnt3a was found to exert an equivalent effect in NIT‐1 β‐cells. In this case, activation of the Wnt/β‐catenin pathway resulted in cross talk with the PI3K/Akt pathway^28^ to promote β‐cell proliferation and to suppress β‐cell apoptosis, resulting in increased islet mass and improved β‐cell function.^29^, ^30^ Although these outcomes are encouraging, it should be noted that the assessments of these proteins were performed in vitro with no evidence of efficacy in vivo. It is also important to consider that neither EPO nor Wnt3a specifically interact with β‐cells, with receptors for both proteins expressed by multiple cell types.^31^, ^32^, ^33^ The administration of either of these proteins may therefore have nonspecific off‐target effects. This risk is already evident for Wnt3a, as the activation of Wnt/β‐catenin signaling initiates and maintains several cancer states,^32^, ^34^ which would likely preclude its value as a treatment for diabetes.

**TABLE 1:** Modulators of PI3K/Akt signaling as putative therapeutics for type 1 diabetes

Modulator	β‐cell effect^a^	References
*Protein and peptide‐based biologics*		
rhEPO	In vitro: Suppressed cytokine‐induced^b^ apoptosis and improved insulin secretion through PI3K/Akt signaling in NIT‐1 β‐cells. Increased expression of downstream Akt target, anti‐apoptotic Bcl‐2	27
Wnt3a	In vitro: Enhanced β‐cell proliferation, improved insulin secretion, and inhibited cytokine‐induced apoptosis via PI3K/Akt and Wnt signaling in NIT‐1 β‐cells	29
GLP‐1	In vitro: Inhibited ROS (hydrogen peroxide)‐induced apoptosis and enhanced cell survival in MIN6 cells, mediated by cAMP‐ and PI3K‐dependent signaling pathway	36
	In vitro: Suppressed methylglyoxal‐induced toxicity, improved mitochondrial function, and inhibited pro‐apoptotic caspase‐3 via PKA and PI3K/Akt signaling in RINm5F, MIN6, and INS‐1 β‐cells	37
	In vivo: Increased β‐cell number in STZ‐induced diabetic rat model, by promoting β‐cell neogenesis through α‐cell transdifferentiation into β‐cells, via regulation of GLP‐1 receptor and downstream transcription factor pathway PI3K/Akt/FOXO1	35
Exendin‐4	In vivo and in vitro: Enhanced β‐cell proliferation in C75BL/6 mice and isolated islets, leading to increased β‐cell mass and number, respectively, in a PI3K/Akt‐dependent manner	39
Liraglutide	In vivo: Restored islet size, prevented apoptosis, and improved nephrin expression (involved in β‐cell survival) in diabetic mice	40
	In vitro: Enhanced β‐cell proliferation, inhibited serum withdrawal‐induced apoptosis, suppressed caspase‐3, and downregulated pro‐apoptotic Bad and FOXO1 in BTC‐6 cells through PI3K/Akt signaling	
Nephrin	In vitro: Stimulated recruitment of PI3K and activated PI3K/Akt signaling, in turn inhibiting downstream Akt substrates pro‐apoptotic Bad and FOXO in mouse islets and BTC‐6 cells	41
GABA	In vivo and in vitro: Enhanced β‐cell proliferation in mouse and human islets through PI3K/mTORC1 pathway. This regenerative effect was amplified by co‐treatment with Ly49, a GABA type A receptor‐positive allosteric modulator, which enhanced β‐cell area and proliferation	43
FhHDM‐1	In vivo: Prevented onset of T1D in NOD mice and increased levels of pancreatic insulin, indicating preservation of β‐cell mass	54
	In vitro: Directly interacted with β‐cells to enhance viability and prevent cytokine‐induced apoptosis without inducing proliferation, through activation of PI3K/Akt pathway	
*Plant extracts*		
Puerarin^c^	In vivo: Suppressed STZ‐induced diabetes and preserved β‐cell mass, inhibited apoptosis, and reversed hyperglycemia in established diabetes. In vitro: Conserved β‐cell viability and insulin secretion following cobalt chloride‐induced apoptosis, mediated by PI3K/Akt signaling. Increased expression of anti‐apoptotic Bcl‐2 in MIN6 β‐cells and primary islets	61
BAI	In vitro: Suppressed TNF‐induced apoptosis, enhanced insulin production, and increased expression of anti‐apoptotic Bcl‐2 and Bcl‐2‐associated X protein in MIN6 β‐cells, in a PI3K/Akt‐dependent manner	62
Saponins^d^	In vitro: Improved INS‐1 cell morphology, viability, and insulin secretion under conditions of glucotoxicity. These effects were associated with increased phosphorylation of Akt and decreased levels of FOXO1	63
C3G	In vivo: Restored normoglycemia after transplantation of C3G‐treated neonatal porcine islets in diabetic mice	64
	In vitro: Inhibited ROS toxicity via PI3K/Akt and extracellular signal‐regulated kinase 1/2 signaling in neonatal porcine islets	
*miRNA*		
miR‐132	In vivo: Increased β‐cell proliferation and survival induced by partial pancreatectomy in mice was associated with enhanced miR‐132 expression, which in turn indirectly activated PI3K signaling via PTEN inhibition	67
	In vitro: Downregulation inhibited MIN6 β‐cell proliferation and increased cleaved caspase‐9. Overexpression generated the opposite effects; it enhanced levels of phosphorylated Akt and downregulated pro‐apoptotic FOXO3	
miR‐17‐92	In vivo: Deletion in mice promoted development of diabetes induced by multiple low doses of STZ, decreased β‐cell number and mass, and increased apoptosis. These effects were associated with suppressed PI3K/Akt signaling due to increased PTEN expression	68
miR‐18	In vitro: Upregulated in MIN6 β‐cells after exposure to pro‐inflammatory cytokine stress and was associated with increased apoptosis and dysregulated insulin secretion. These effects were attributed to miR‐18‐induced repression of a component within the PI3K/Akt pathway (neuron navigator 1), thus downregulating pAkt and PI3K. Knockdown of miR‐18 produced the opposite results	69
miR‐139‐5p	In vivo: Overexpression of miR‐139‐5p in mice downregulated expression of PICK1, which normally protects β‐cells via PI3K/Akt activation	70
	In vitro: Negative regulation by miR‐139‐5p of PICK1 repressed PICK1‐mediated activation of PI3K/Akt signaling. Overexpression of PICK1 protected β‐cells from glucotoxicity via PI3K/Akt activation	
miR‐122	In vitro: Inhibition of miR‐122 suppressed oxidative stress and apoptosis induced by STZ in INS‐1 β‐cells, via activation of PI3K/Akt signaling	71
let‐7	In vivo and in vitro: Biogenesis of let‐7 miRNA (PI3K/Akt suppressor) was reduced by protein Lin28a. Reduction of let‐7 biogenesis via Lin28a overexpression resulted in activation of PI3K/Akt and protected β‐cells from STZ‐induced destruction in mice and MIN6 cells	72

Abbreviations: Bad, Bcl‐2‐associated death promoter; BAI, baicalin derived from *Scutellaria baicalensis*; C3G, cyanidin‐3‐O‐glucoside derived from anthocyanin; FhHDM‐1; *Fasciola hepatica* helminth defense molecule 1; FOXO1 and FOXO3, forkhead box proteins O1 and O3; GABA; γ‐aminobutyric acid; GLP‐1, glucagon‐like peptide 1; miRNA, microRNA; mTORC1, mammalian target of rapamycin complex 1; NOD, nonobese diabetic; PI3K, phosphoinositide 3‐kinase; PICK1, protein interacting with C‐kinase 1; PTEN, phosphatase and tensin homolog; rhEPO; recombinant human erythropoietin; ROS, reactive oxygen species; STZ, streptozotocin; T1D, type 1 diabetes; TNF, tumor necrosis factor; Wnt3a, protein that promotes the activation of PI3K/Akt signaling with cross talk with Wnt signaling.

^a^ Various β‐cell lines have been investigated in the different studies, including murine BTC‐6, NIT‐1, MIN6, INS‐1, and the rat β‐cell line RINm5F.

^b^ The most commonly used cytokine mix to induce β‐cell apoptosis combines interleukin‐1 β, TFN, and interferon γ, all of which have been shown to act synergistically to induce β‐cell apoptosis.

^c^ Extracted from Radix puerariae.

^d^ Derived from *Momordica charantia*.

Circumventing the issue of cell specificity, is the exploitation of glucagon‐like‐peptide‐1 (GLP‐1), as this interacts with a receptor that is only expressed on β‐cells (and neurons). GLP‐1 is a gut‐derived incretin that is naturally produced in response to food ingestion to enhance insulin secretion. Specifically, GLP‐1 has been reported to promote proliferation and inhibit apoptosis of β‐cells, in vivo and in vitro, and to attenuate apoptosis induced by several pro‐apoptotic agents (such as STZ and reactive oxygen species [ROS]).^35^, ^36^ Chang et al (2016) showed that GLP‐1 suppressed methylglyoxal (MG)‐induced apoptosis in a rat insulinoma cell line (RINm5F) by improving mitochondrial function and activating PKA and PI3K/Akt signaling, thereby causing Akt phosphorylation and subsequent inhibition of the cleavage (ie, activation) of pro‐apoptotic caspase‐3. These effects were corroborated by experiments using MIN6 and INS‐1 β‐cells.^37^ In addition to enhancing proliferation and survival, GLP‐1 has been recently shown to putatively promote β‐cell neogenesis in a severe insulin‐deficient diabetic rat model induced by administration of a single high dose of STZ. Indeed, the consequent increase in β‐cell number following GLP‐1 treatment was not attributable to β‐cell replication but rather to α‐cell dedifferentiation and subsequent transdifferentiation into glucose‐responsive insulin‐secreting β‐cells. This was associated with the regulation of the GLP‐1 receptor and its downstream transcription factor pathway PI3K/Akt/FOXO1.^35^

While these beneficial effects of GLP‐1 strongly support its use as a therapeutic agent, it is rapidly degraded in vivo (t_1/2_ ~ 2 minutes) by the endogenous enzyme dipeptidyl‐peptidase‐IV (DPP‐4).^38^ To extend bioavailability to improve clinical efficacy, several synthetic GLP‐1 receptor agonists have been developed. For instance, the native molecule exendin‐4 has been shown to enhance β‐cell proliferation and inhibit apoptosis, both in vitro and in vivo, thereby increasing β‐cell number and mass, respectively. These effects were abolished in the presence of the PI3K inhibitor LY294002, establishing that the pro‐survival/proliferation effect was mediated by PI3K/Akt signaling in β‐cells.^39^ Likewise, the human GLP‐1 analogue liraglutide has been shown to promote β‐cell proliferation and inhibit apoptosis, both in vitro and in vivo. Indeed, in BTC‐6 β‐cells, liraglutide suppressed apoptosis induced by serum withdrawal through PI3K/Akt phosphorylation, leading to the inhibition of caspase‐3 activity via a mechanism like that of GLP‐1. The downstream Akt targets, pro‐apoptotic Bad and FOXO1 transcription factor, also underwent inhibitory phosphorylation. These observations were corroborated in an animal model of overt diabetes where liraglutide restored islet size, ameliorated β‐cell apoptosis, and increased expression levels of nephrin,^40^ a key protein involved in β‐cell survival signaling .^41^ Despite the positive effects on β‐cell survival in vivo, there are some concerns regarding the safety profile of these GLP‐1 analogues due to their ability to enhance the proliferation of cells. While a consensus has not been reached, evidence from some clinical studies has shown an expansion of exocrine and endocrine pancreatic cells with a possible association to pancreatic cancer.^42^

As an alternative biologic, γ‐aminobutyric acid (GABA) may be a candidate therapeutic agent. It is endogenously produced in β‐cells and has been found to stimulate β‐cell proliferation in mouse and human islets through the PI3K/mTORC1 pathway. Co‐treatment of mice with both GABA and Ly49, a novel GABA type A (GABA_A_) receptor‐positive allosteric modulator, amplified these positive effects. Furthermore, co‐treatment with GABA and Ly49 increased β‐cell area and proliferation, as compared to mice treated with GABA alone, and similar observations were made using human islets.^43^ Importantly, a number of GABA_A_ receptor agonists are currently in clinical use as a treatment for epilepsy,^44^ which suggests that they are considered safe for use in humans and so could potentially be tested in clinical trials as a treatment for T1D.

### 5.2 The secreted products of parasitic worms

Numerous epidemiological studies and experimental investigations have now clearly demonstrated an inverse relationship between infection with parasitic worms (helminths) and the incidence of autoimmune diseases, such as T1D.^45^, ^46^ The beneficial effect of a parasite infection in preventing autoimmunity has been explained by the “old friends” hypothesis”.^47^ This postulates that because the normal protective immune response mounted in response to pathogens is largely inefficient against large multicellular worms, humans have evolved, over millennia of co‐existence, to tolerate the presence of helminths. This outcome is supported by the potent modulation of human immune responses by the parasites. The net result is the suppression of antimicrobial pro‐inflammatory responses, which are of the same phenotype associated with the development of T1D (ie, pronounced TH1 and TH17 immune responses).^48^ Testament to this, the development of T1D may indeed be driven by some forms of bacteria.^49^ Accordingly, the removal of helminth exposure, due to improved hygiene and medical practices, has been associated with an increased risk of inappropriate immune responsiveness to autoantigens and the development of autoimmunity.^48^ Therefore, controlled helminth parasite infection or exposure to their excretory/secretory products has been investigated as a therapeutic strategy to prevent and/or ameliorate immune‐mediated diseases.^50^, ^51^

With the assumption that the capacity for helminths to modulate host immune responses is the primary mechanism by which these parasites are preventing disease, helminth secretions have been specifically mined for immune‐modulatory molecules to be used as therapeutic modalities. While several agents have been identified,^51^ we have recently discovered that the helminth defense molecule 1 secreted by the liver fluke *Fasciola hepatica* (FhHDM‐1) has a unique capacity to directly target β‐cells. This presents an opportunity for the development of a novel therapeutic for treating diabetes that has been pharmacologically optimized through the co‐evolution of a parasitic worm and its human hosts.

Administration of FhHDM‐1 via intraperitoneal injection prevented the onset of T1D in NOD mice.^52^, ^53^ Importantly, only a short‐course of FhHDM‐1 treatment was required, with mice presenting as nondiabetic up to 26 weeks after the final injection (experimental endpoint). While the half‐life of FhHDM‐1 has not been determined, this outcome would suggest that the biological effect is long‐lasting, which provides a positive pharmacological comparison to GLP and its analogues. Disease protection in FhHDM‐1‐treated mice was associated with decreased insulitis (likely indicative of decreased β‐cell apoptotic rates and hence autoantigen generation) and increased levels of pancreatic insulin (suggesting that FhHDM‐1 preserved β‐cell mass against an aggressive autoimmune background) as compared to vehicle controls. Further, biodistribution studies showed that FhHDM‐1 localized to the pancreas and in vitro FhHDM‐1 directly interacted with β‐cells, of both murine and human origin, to enhance viability and prevent apoptosis without inducing β‐cell proliferation. These positive effects on the β‐cells were associated with the activation of PI3K/Akt signaling, as revealed by proteomics and RNAseq analysis. The inhibition of the PI3K/Akt pathway reversed the protective effects of FhHDM‐1 on β‐cells, confirming a functional role for this pathway in mediating the positive effect of the peptide.^54^

Interrogation of numerous parasite and mammalian genomes has established that the helminth defense molecule peptide family is unique to flatworms,^55^, ^56^ suggesting an adaptation for a specific biological function while they reside within their mammalian hosts. Testament to this host‐parasite relationship, FhHDM‐1 is not cytotoxic to mammalian cells,^57^ and it does not induce any adverse activity in a broad range of clinically relevant pharmacology assays (unpublished data). Therefore, FhHDM‐1 is highly selective and efficacious in its ability to preserve β‐cell mass without inducing proliferation while simultaneously being safe and well tolerated. Collectively, these properties make FhHDM‐1 an innovative candidate for maintaining β‐cell survival and function against the inflammatory backgrounds underlying T1D.

### 5.3 Plant extracts

The healing properties of medicinal herbs has been applied for therapeutic intervention since the dawn of human history. Today, hundreds of plants have been reported to exert positive effects for the treatment of diabetes by reducing blood glucose levels and decreasing the occurrence of the complications caused by hyperglycemia.^58^, ^59^, ^60^ However, there remains a lack of detailed information regarding the specific bioactive component(s) and the exact mechanism(s) of action through which many of these plants exert their therapeutic effect. Of the plant extracts that have been identified and tested specifically in models of T1D to date, seven have been shown to exert their therapeutic effects via PI3K/Akt‐mediated modulation of β‐cell function (Table 1).^61^, ^62^, ^63^, ^64^ All these compounds commonly protected β‐cells from cytotoxic stimuli (in vitro, pro‐inflammatory cytokines^61^, ^62^ or high glucose^63^ and in vivo, pro‐inflammatory cytokines^61^ or ROS^64^). In all cases, apoptosis was actively inhibited, β‐cells retained viability, and glucose‐stimulated insulin secretion was restored.

### 5.4 Modulation of microRNA expression

MicroRNA (miRNA) are short, noncoding RNA that are recognized as major regulators of gene expression through posttranscriptional mechanisms, thereby indirectly modulating diverse physiological and pathological processes.^65^ Although miRNA therapeutics have not yet translated as approved therapies, many are in clinical trials, and the first small‐interfering RNA was recently granted Food and Drug Administration (FDA) approval, which signals the emergence of this therapeutic approach.^66^ There is evidence of clinical application for both miRNA mimics (to target gene expression) as well as miRNA inhibitors (to enhance gene expression).

An exploration of the literature unsurprisingly reveals several miRNA that directly or indirectly modulate PI3K/Akt signaling to exert positive effects on β‐cell survival and function under conditions akin to T1D development (Table 1). Enhanced β‐cell proliferation, induced by partial pancreatectomy in mice, was associated with an upregulation of miR‐132 expression, which in turn indirectly activated PI3K/Akt signaling through inhibition of PTEN. Conversely, deletion of miR‐132 in vivo inhibited β‐cell proliferation. These findings were corroborated in vitro using MIN6 β‐cells, wherein downregulation of miR‐132 suppressed proliferation while concomitantly increasing levels of cleaved (apoptotic) caspase‐9. On the other hand, overexpression of miR‐132 produced the opposite effect through inhibition of PTEN, subsequent upregulation of phosphorylated Akt levels, and thus downregulation of its pro‐apoptotic substrate FOXO3. Therefore, targeting the miRNA‐mediated activation of PTEN/Akt signaling may serve as a therapeutic avenue to positively modulate β‐cell mass.^67^ This notion is further supported by the observation that β‐cell‐specific deletion of miR‐17‐92 in vivo promoted the development of experimental autoimmune diabetes induced by multiple low doses of STZ, characterized by elevated fasting blood glucose levels and impaired glucose tolerance. These outcomes were attributable to reduced β‐cell number and mass and an increased incidence of β‐cell apoptosis. In turn, these deleterious effects were associated with higher levels of PTEN expression, which suppressed PI3K/Akt signaling .^68^

Conversely, the inhibition of specific miRNA can enhance PI3K/Akt signaling to confer β‐cell survival. For instance, under pro‐inflammatory conditions induced by IL‐1β treatment, MIN6 β‐cells showed significant upregulation of miR‐18 expression levels, which was associated with an increased incidence of apoptosis, and dysregulated insulin production and secretion in response to glucose. These deleterious effects on β‐cell survival and function were associated with miR‐18‐induced repression of neuron navigator 1 (NAV1), a constituent of the PI3K/Akt pathway, resulting in the downregulation of pAkt and PI3K expression levels. Importantly, the knockdown of miR‐18 generated the opposite outcomes, suggesting that miR‐18 can be inhibited to promote PI3K/Akt signaling and consequently ameliorate β‐cell dysfunction and apoptosis.^69^ Similarly, overexpression of miR‐139‐5p negatively regulated the expression levels of protein interacting with C‐kinase 1 (PICK1), which normally provides functional protection of β‐cells through PI3K/Akt activation.^70^ Likewise, although their specific gene targets were not investigated, the inhibition of miR‐122^71^ or let‐7 miRNA^72^ resulted in the activation of PI3K/Akt signaling in β‐cells, which mediated their protection from STZ‐induced destruction in vitro^71^, ^72^ and in vivo.^72^

Collectively, the aforementioned studies indicate that upregulation of PI3K/Akt signaling through dialogue with various miRNA can exert positive effects on β‐cell function and survival, and, as such, these miRNA represent viable therapeutics/targets for the treatment of T1D.

## 6 MODULATION OF PI3K/AKT in β‐cells has applications for treatment of T2D and islet transplantation

**FIGURE 2:**
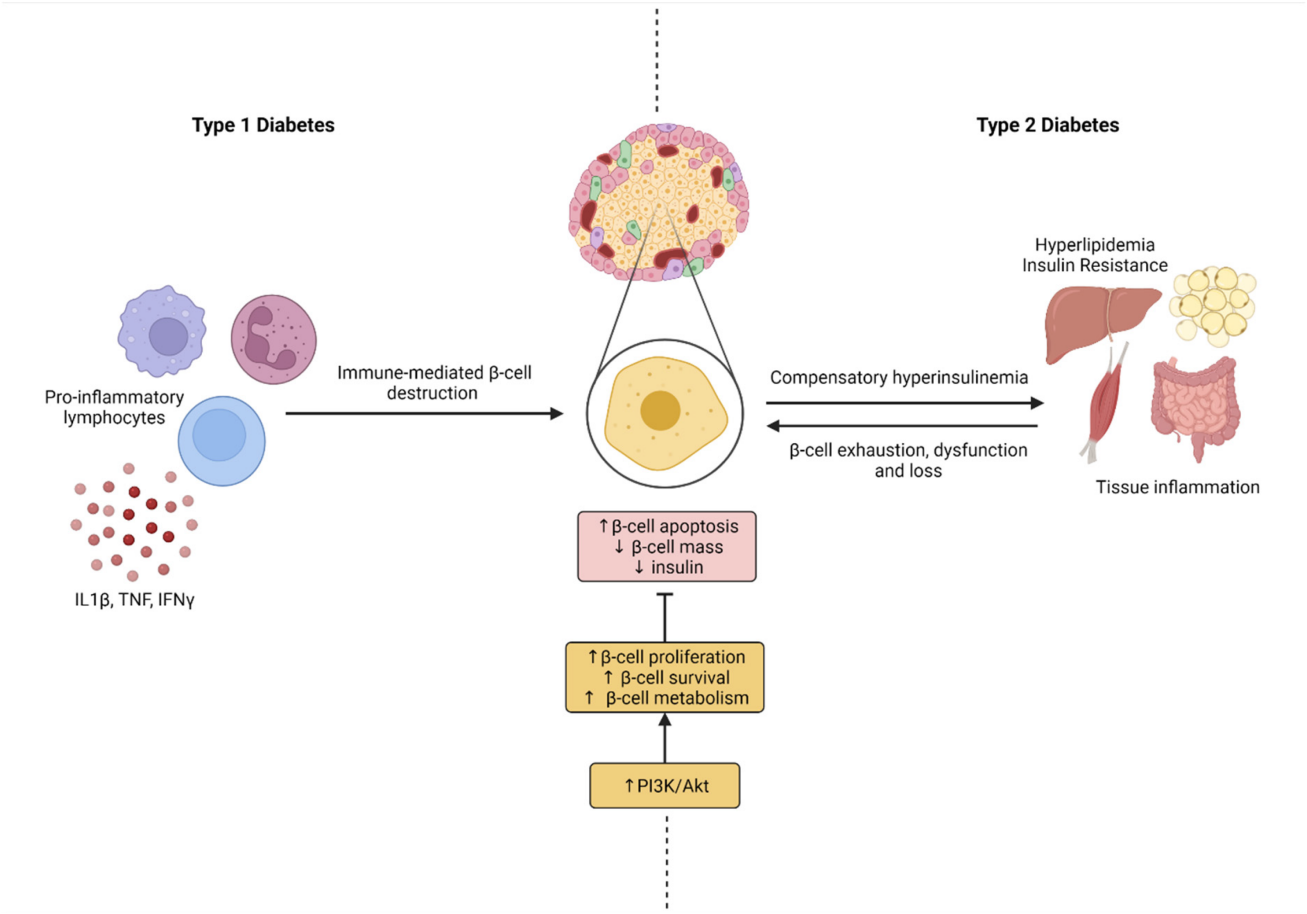
The pathogenesis of type 1 (T1D) and type 2 diabetes (T2D) both ultimately lead to the decline of β‐cell mass and function. Modulation of PI3K/Akt signalling can act as a therapeutic strategy to counteract β‐cell loss. In T1D, autoreactive lymphocytes and pro‐inflammatory cytokines (such as IL1β, TNF and IFNγ) drive β‐cell destruction. In T2D, β‐cells undergo compensatory expansion and increased insulin secretion in response to hyperlipidaemia and insulin resistance in the tissues, thus causing β‐cell exhaustion and death. In both conditions, there is a decrease in β‐cell mass and insulin secretion. This decline can be counteracted by activation of the PI3K/Akt pathway, which has been shown to promote β‐cell proliferation, survival and metabolism. Created with Biorender.com

While their etiopathologies are different, T1D and T2D are both pro‐inflammatory disorders characterized by an ultimate decline in β‐cell mass and function. In T1D, β‐cell loss is mediated by a sustained process of autoimmune destruction that occurs over several months/years, such that at diagnosis β‐cell mass is reduced by 80% to 90%. In T2D, β‐cells undergo a compensatory expansion in response to insulin resistance, thereby causing β‐cell exhaustion and death. Furthermore, genome‐wide association studies of T2D show that most genes are modulators of β‐cell mass and/or function. In addition, preservation of β‐cell mass during islet preparation and after islet transplantation is critical and represents a major clinical hurdle to widespread application for patients. Therefore, approaches to preserve functional β‐cell mass offers therapeutic potential for both T1D and T2D (Figure 2).^73^

Many of the therapeutic strategies targeting the PI3K/Akt pathway in β‐cells that are described above for the treatment of T1D have also been tested and in some instances utilized as T2D therapeutics because they modulate β‐cell function to enhance glucose responsiveness (Table 2). While they are efficacious and generally well tolerated, these treatments are limited by several shortcomings. As mentioned, GLP‐1 analogues are hindered by a very short half‐life,^38^ adverse effects (the most common being gastrointestinal and injection site reactions),^74^ and potential associations with pancreatic cancer due to the induction of cellular proliferation.^42^ Metformin is an alternatively prescribed treatment for T2D, which has been shown to inhibit endoplasmic reticulum stress, dysfunction, and apoptosis in NIT‐1 cells via PI3K/Akt signaling.^75^ However, metformin is contraindicated in patients with risk factors for lactic acidosis and has common side effects, such as gastrointestinal intolerance, although they are usually transient.^76^ Thus, there is a continued clinical need for alternative therapeutic strategies. Like the reported efficacy in T1D, several plant extracts, proteins/peptides, and miRNA have been identified with potential antidiabetic effects in T2D. These agents have been shown to protect β‐cells from injury and apoptosis in vivo, such as in high‐fat‐diet (HFD)^77^ and HFD/STZ^78^, ^79^, ^80^ murine models, and in vitro under pro‐inflammatory conditions following exposure to STZ^81^ or inducers of oxidative^82^, ^83^ and endoplasmic reticulum stress.^75^ They also exert beneficial β‐cell effects under basal conditions by enhancing glucose‐stimulated insulin secretion.^84^ All these positive effects were mediated by regulation of PI3K/Akt signaling and its associated pathways.

**TABLE 2:** Modulators of PI3K/Akt signaling as putative therapeutics for type 2 diabetes

Modulator	β‐cell effect	References
*Plant extracts*		
Hydroxysafflor yellow A	In vivo: Protected β‐cells from inflammatory damage and apoptosis through activation of PI3K/Akt signaling in T2D rats induced by HFD and low‐dose STZ	78
Jiaogulan tea and white tea	In vivo: Ameliorated T2D in C57BL/6 mice exposed to HFD/STZ and protected β‐cells against oxidative and inflammatory damage mediated through AMPK/PI3K pathway	79
Vin‐C01 and Vin‐F03	In vitro: Promoted β‐cell survival and inhibited STZ‐induced apoptosis in INS‐1 cells via regulation of IRS‐2/PI3K/Akt signaling pathway	81
Methyl caffeate	In vitro: Enhanced glucose‐stimulated insulin secretion and activation of IRS‐2, PI3K, and Akt proteins in INS‐1 cells	84
Banxia xiexin	In vitro: Suppressed tert‐butyl hydroperoxide‐induced apoptosis and improved insulin secretion through regulation of PI3K/Akt signaling and FOXO1 in MIN6 cells	82
*Proteins*		
Irisin	In vivo and in vitro: Attenuated lipotoxicity‐induced β‐cell insulin resistance and inflammatory response in HFD C57BL/6J mice and MIN6 cells via activation of PI3K/Akt/FOXO1 signaling	77
Sericin	In vivo: Ameliorated HFD/STZ induced islet damage and improved β‐cell function through enhanced PI3K/Akt signaling	80
Metformin	In vitro: Inhibited endoplasmic reticulum stress, dysfunction, and apoptosis in NIT‐1 cells via AMPK and PI3K/Akt signaling	75
*miRNA*		
miR‐126	In vitro: Resveratrol‐induced upregulation of miR‐126 alleviated uric acid‐induced injury and apoptosis in MIN6 cells through the activation of PI3K/Akt signaling	83

Abbreviations: AMPK, adenosine monophosphate‐activated protein kinase; FOXO1, forkhead box protein O1; HFD, high‐fat diet; IRS‐2, insulin receptor substrate 2; miRNA, microRNA; PI3K, phosphoinositide 3‐kinase; STZ, streptozotocin; T2D, type 2 diabetes.

## 7 CONCLUSION

PI3K/Akt signaling represents a promising therapeutic target for diabetes. Modulation of the pathway, singularly or through miRNA interactions, will aid in regulating the β‐cell processes that determine the fate of β‐cell mass. For instance, upregulation of PI3K/Akt signaling can promote β‐cell function, survival, and/or proliferation, thereby counteracting the disproportionately large waves of apoptosis during neonatal pancreatic remodeling at a time concurrent with the initiation of autoimmunity. Likewise, β‐cell mass lost from autoimmune destruction may be restored or maintained by PI3K/Akt‐mediated stimulation of β‐cell survival, metabolic, and anti‐apoptotic pathways. All these activities are crucial not only in T1D, but also in the context of T2D, which is now recognized as an inflammatory disease in which β‐cell mass and function need to be preserved. Indeed, modulation of PI3K/Akt signaling can support exhausted β‐cells to alleviate hyperglycemia and insulin resistance. Therapeutic applications of enhancing PI3K/Akt signaling in β‐cells could also be extended to islet transplantation, to counteract the large amount of β‐cell death that occurs throughout the process of isolation and delivery, and then post transplant against allogeneic and autoimmune destruction.

## DISCLOSURES

The authors declare no conflict of interest.
